# Dietary Patterns in Association With Hypertension: A Community-Based Study in Eastern China

**DOI:** 10.3389/fnut.2022.926390

**Published:** 2022-07-08

**Authors:** Cuicui Wang, Yanmin Zheng, Ya Zhang, Dong Liu, Li Guo, Bo Wang, Hui Zuo

**Affiliations:** ^1^School of Public Health, Medical College of Soochow University, Suzhou, China; ^2^Jiangsu Key Laboratory of Preventive and Translational Medicine for Geriatric Diseases, Medical College of Soochow University, Suzhou, China; ^3^Department of Nutrition and Food Hygiene, Suzhou Center for Disease Control and Prevention, Suzhou, China; ^4^Department of Disease Prevention and Health Care, Soochow University Hospital, Soochow University, Suzhou, China

**Keywords:** dietary pattern, factor analysis, hypertension, dietary recall, cross-sectional study

## Abstract

**Objective:**

This study aimed to explore the association between dietary patterns and hypertension based on a community–based survey in Suzhou, Eastern China.

**Methods:**

This cross–sectional analysis was undertaken from the subset of the Suzhou Food Consumption and Health State Survey in 2018–2019. Adults aged ≥ 18 years were invited to participate in this survey. Dietary intake was collected by a 24–h dietary recall and a weighing method over three consecutive days (including two weekdays and one weekend day). Dietary patterns were defined using factor analysis. Association between the dietary patterns and hypertension was examined by multivariable logistic regression models with adjustment for covariates. Moreover, sensitivity analysis was used to reinforce our findings.

**Results:**

A total of 2,718 participants were included in the final analysis. Rice-vegetable pattern, fast food pattern, fruit-dairy pattern, and wheat-meat pattern were identified. We observed that the fruit-dairy pattern was inversely associated with hypertension after adjustment for all the covariates (OR = 0.55; 95% CI: 0.40, 0.75; *P* = 0.002). The association between the wheat-meat pattern and hypertension was attenuated and became statistically nonsignificant in sensitivity analyses. The other two patterns were not significantly associated with hypertension (*P* > 0.05).

**Conclusion:**

The fruit-dairy pattern was inversely associated with the risk of hypertension among Chinese adults. Our findings further emphasize the important role of optimal diet combination in the prevention of hypertension.

## Introduction

Hypertension is one of the major risk factors affecting the global burden of disease and is one of the most important risk factors for cardiovascular disease (1). It was reported that a 10 mmHg reduction in systolic blood pressure (BP) could lower the risk of major stroke by 27%, heart failure by 28%, and cardiovascular disease events by 20% (2). Therefore, the primary prevention of hypertension has now become a top priority for global public health. Furthermore, hypertension is the leading modifiable risk factor for cardiovascular disease, the top cause of death in China (3). In China, five waves of nationwide hypertension surveys have been conducted since the 1950s (1959, 1979, 1991, 2002, and 2012, respectively). Based on the survey data, the prevalence of hypertension continuously increased from 5.11% in 1959 to 23.2% in 2012 among adults (4).

Accumulating evidence has suggested that diet plays a significant role in the development and progression of hypertension (5–7). Dietary pattern was more comprehensive to reflect the synthesized effect of foods or nutrients compared with individual food or nutrient (8). It has been widely used as an alternative method to assess the relationship between whole diet and hypertension (9). Over the past few decades, dietary patterns, such as Dietary Approaches to Stop Hypertension (DASH) (10) and Mediterranean Dietary Pattern (MDP) (11), have been suggested to have a protective effect on hypertension.

With the rapid development of China's economy in recent years, China has undergone a rapid nutritional transition. Meanwhile, due to China's vast territory and rich food variety, dietary patterns may vary between different regions and cultural environments. For example, in North China, the diet in Inner Mongolia was characterized by “high protein,” “traditional northern,” “modern,” and “condiments” patterns (12). In Southwest China, Diqing of Yunnan Province, three dietary patterns were identified, namely, “Grassland healthy,” “Tuber and meat,” and “Fruit and vegetable” (13). In Yangtze River Delta region, people tended to adhere to a healthy diet pattern named as “Southern River-style dietary pattern,” including high consumption of vegetables and fruits in season, freshwater fish and shrimp, and legumes, and moderate consumption of whole-grain rice, plant oils (mainly rapeseed oil), and red meat (14). However, there were limited studies on the association between dietary patterns and hypertension in Eastern China (15). Moreover, high economic development in Eastern China may be accompanied by high levels of environmental pollution and corresponding food contaminants (16, 17). Hence, the aim of this study was, therefore, to provide the latest evidence on the association between dietary patterns and hypertension among community residents in Eastern China.

## Methods

### Participants and Setting

**Figure 1:**
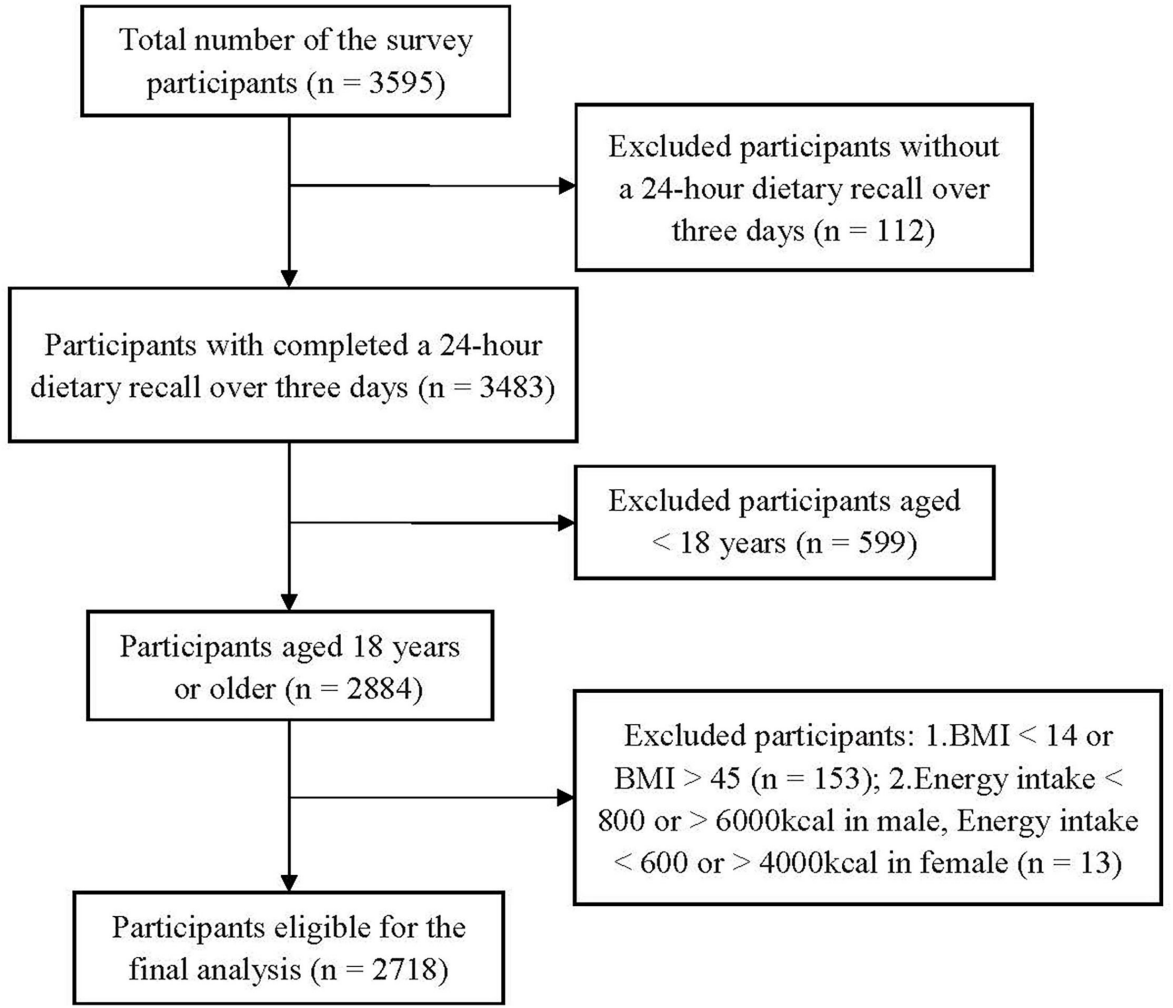
Flowchart of study selection.

The subset of the Suzhou Food Consumption and Health State Survey was a cross-sectional study conducted in Suzhou, Eastern China, in 2018–2019. A multistage stratified cluster random sampling method was used to recruit potential participants. A total of 3,595 participants were invited. Among them, 112 participants did not complete the dietary survey. We excluded 599 participants under the age of 18 years and those with implausible dietary data (energy intake <800 or > 6,000 kcal in males, energy intake <600 or >4,000 kcal in females) (*n* = 13). In addition, we excluded those participants with BMI <14 kg/m^2^ or BMI > 45 kg/m^2^ (n = 153). A total of 2,718 participants (males =1,288, females = 1,430) were included in the final analysis (Figure 1).

### Dietary Assessment

Dietary data were collected using a 24-h dietary recall and a weighing method over 3 consecutive days (including 2 weekdays and 1 weekend day) by trained investigators. Participants were asked to recall the types and quantities of food consumed during a 24-h dietary recall *via* a face-to-face interview. A weighing method was used to collect the information on daily consumption of major seasonings, including cooking oil, salt, sugar, monosodium glutamate, soy sauce, and vinegar. This method has been used in the China National Nutrition Surveys and is widely accepted by international researchers (18). This was done by interviewers at the participants' homes after dinner at night. Consumption of condiments was obtained by weighing condiments purchased and wasted. The survey was conducted following a standardized protocol.

### Definition of Hypertension

Data on hypertension was based on a measurement of BP and self-report *via* a questionnaire. BP was measured using an electronic sphygmomanometer (Omron Hp-1300, OMRON Corporation, Japan) on the right arm positioned at heart level after a 5-min rest period in a sitting position following standard procedures. BP was measured two times at 2-min intervals, and the mean value of two measurements was used for data analysis. Meanwhile, participants were asked the following questions: “Have you been diagnosed with hypertension by a qualified doctor in a township or higher level hospital?” and “Have you taken any antihypertensive medicine within the last 2 weeks?” Participants were considered as hypertensive if they had a systolic BP ≥ 140 mmHg, diastolic BP ≥ 90 mmHg, diagnosis of hypertension, or antihypertensive medication.

### Covariates

The participants' information, including age, gender, education, smoking status, alcohol drinking, sleeping disorders, and family history of hypertension, were collected by a reviewer-administrated questionnaire at enrollment. Education was classified into three categories, namely, low: illiterate/primary school; medium: junior middle school; and high: senior middle school/university. Smoking status was categorized into three groups, namely, non-smokers, ex-smokers, and current smokers. Participants were defined as drinking alcohol if the average drinking frequency was ≥ once a month in the last 12 months (19). Participants were considered as having sleep disorders if they had self-reported sleep-disordered breathing or insomnia (20). Participants were asked the following question: “How much time do you usually spend sitting every day? (including all sedentary time, such as working, studying, reading, watching TV, using computer, and taking rest whenever sitting). Sedentary time was divided into tertiles. The weighing method over 3 consecutive days (including 2 weekdays and 1 weekend day) in the population was performed to estimate the salt intake as described in a recent study (21). Daily salt intake was divided into three categories, namely, <4 g/day, 4–6 g/day, and >6 g/day.

Self-reported history of diabetes, dyslipidemia, coronary heart disease, stroke (ischemic and hemorrhagic stroke), and other chronic diseases [including chronic obstructive pulmonary disease, asthma, bone and joint disease, and neck or waist diseases (such as cervical spondylopathy, lumbar strain, and spinal disc herniation), chronic digestive system disease, chronic urinary system disease, and malignant tumor] ever diagnosed in a township or higher-level hospital by a qualified doctor were recorded.

Waist circumference (WC) was measured midway between the lowest rib and the iliac crest or 1 cm above the umbilicus, against bare skin, but subtracting 1 cm if on top of undergarments (22). WC was continuously measured two times to the nearest 0.1 cm using a soft non-stretchable tape, and the average of two measurements was used for data analysis. The waist circumference threshold for abdominal obesity was ≥ 85 cm in women and ≥ 90 cm in men, respectively (23).

### Statistical Analysis

The sociodemographic and lifestyle characteristics were described as medians (interquartile ranges) for continuous variables and frequencies with percentages for categorical variables. The chi-square test was used to compare the characteristics of participants with and without hypertension for categorical variables, and Kruskal-Wallis tests were used for continuous variables.

We grouped food items according to similar nutrient contents. Accordingly, all of the foods were classified into 21 groups, including rice and products, vegetables (fresh leafy and non-leafy vegetables), aquatic products, red meat, other crops and potatoes, eggs, soybeans and products, phycomycetes (mushroom, *Auricularia auricula, Porphyra*, and kelp), nuts, poultry, fast food, snacks, beverages, dairy, fruit, yogurt, sweets, desserts, pickles, wheat and wheat products, and alcohol. Dietary patterns were identified by factor analysis. The number of factors retained was determined using the scree plot (eigenvalues > 1) and the interpretability of every factor. Orthogonal (varimax) rotation was applied to improve interpretability and minimize the correlation between the factors. As a result, four dietary patterns were extracted, and dietary pattern scores were categorized into quartiles.

Association between each dietary pattern and hypertension was tested by binary logistic regression with adjustment for potential confounding factors. Three sensitivity analyses were performed to determine the robustness of findings in the primary analysis. First, we restricted the risk-association analyses to those participants without self-reported diabetes, dyslipidemia, coronary heart disease, stroke, and other chronic diseases. Second, we analyzed the risk associations among participants without self-reported hypertension to minimize the possibility of reverse causation. Third, we further examined the risk associations by taking the participants with prehypertension [defined as systolic BP ≥ 120 mmHg and <140 mmHg or diastolic BP ≥ 80 mmHg and <90 mmHg (24)] as a separate group using ordinal logistic regression models. All statistical tests were two-tailed, and a *P*-value <0.05 was considered statistically significant. All statistical analyses were performed using SAS statistical software (version 9.4; SAS Institute, Inc.).

## Results

### Participant Characteristics

Of the 2,718 eligible participants, 727 participants reported with hypertension. Compared to those without hypertension, individuals with hypertension were generally older and had abdominal obesity (*P* <0.001). The participants with hypertension were more likely to be men, current smoker, alcohol drinker, and have high education level (*P* <0.001). In addition, there were significant differences in energy intake, sleeping disorders, family history of hypertension, daily salt intake, and sedentary time (*P* <0.05) between the participants with and without hypertension (Table 1).

**Table 1:** Sociodemographic and lifestyle characteristics between participants with and without hypertension: The Suzhou Food Consumption and Health Survey.

	**Hypertension** ***(n* = 727)**	**No hypertension** **(*n* = 1991)**	***P-trend***
Age, yrs	63 (54, 69)	40 (33,35)	<0.001
Male (%)	385 (53.0)	903 (45.4)	<0.001
Education (%)			<0.001
Low	178 (24.6)	237 (12.0)	
Medium	229 (31.6)	379 (19.1)	
High	317 (43.8)	1,367 (68.9)	
Smoking (%)			<0.001
Never smoker	462 (63.5)	1,543 (77.9)	
Ex-smoker	85 (11.7)	77 (3.9)	
Current smoker	180 (24.8)	361 (18.2)	
Alcohol drinking (%)			<0.001
Yes	271 (37.4)	577 (29.2)	
No	453 (62.6)	1,397 (70.8)	
Sleeping disorders (%)			<0.001
Yes	446 (61.5)	700 (35.8)	
No	279 (38.5)	1,258 (64.2)	
WC (%)			<0.001
Normal	302 (41.5)	1,311 (65.8)	
Abdominal obesity	425 (58.5)	680 (34.2)	
Family history of hypertension (%)			<0.001
Yes	162 (22.3)	842 (42.3)	
No	458 (63.0)	842 (42.3)	
Unknown	107 (14.7)	307 (15.4)	
Daily salt intake (g/d)			0.244
<4	270 (37.1)	675 (33.9)	
4–6	196 (27.0)	543 (27.3)	
> 6	261 (35.9)	773 (38.8)	
Sedentary time (h/d)			0.001
<3	300 (41.3)	701 (35.2)	
3–6	271 (37.3)	727 (36.5)	
> 6	156 (21.4)	563 (28.3)	
Energy intake (kcal/d)	1,546.9 (1,194.7, 2,006.8)	1,605.6 (1,251.1, 2,076.4)	0.037

*WC, waist circumference. Abdominal obesity was defined as waist circumference ≥ 85 cm and ≤ 90 cm for women and men, respectively. Data are presented as median (p25, p75) for continuous measures and n (%) for categorical measures. P-values were determined by chi-square tests for categorical variables and Kruskal-Wallis tests for continuous variables*.

### Dietary Patterns

Four dietary patterns were identified, namely, the rice-vegetable pattern, the fast food pattern, the fruit-dairy pattern, and the wheat-meat pattern, respectively, based on their main food components. The rice-vegetable pattern was characterized by a high intake of vegetables, rice and products, and aquatic products. The fast food dietary pattern was highly correlated with intakes of fast food, beverages, soybean, and products. The fruit-dairy dietary pattern was related to high intakes of fruits, yogurt, and milk. The wheat-meat pattern was distinguished by high intakes of wheat, red meat, and desserts. The four dietary patterns accounted for 28.5% of the variance in the total food intake, as shown in Table 2.

**Table 2:** Factor loading of dietary patterns.

	**Rice-vegetable**	**Fast food**	**Fruit-dairy**	**Wheat-meat**
Rice and products	0.59	–	–	–
Vegetables	0.69	–	–	–
Aquatic products	0.57	–	–	–
Red meat	0.40	–	–	0.56
Other crops and potatoes	0.28	–	0.31	–
Eggs	0.36	–	0.20	0.23
Soybean and products	0.27	0.41	–	–
Phycomycete	0.30	0.39	–	–
Nuts	0.28	0.36	–	–
Poultry	0.41	0.32	–	–
Fast food	−0.23	0.59	–	–
Snacks	–	0.38	–	–
Beverages	–	0.45	–	–
dairy	–	0.22	0.44	–
Fruit	–	–	0.63	–
Yogurt	–	–	0.45	–
Sweets	–	–	0.23	–
Desserts	–	–	0.20	−0.34
Pickles	–	–	−0.35	−0.31
Wheat and products	–	–	–	0.62
Alcohol	–	–	–	−0.31
Variance explained (%)	10.3	6.53	6.01	5.63

*Absolute values <0.20 are indicated by a dash for simplicity*.

### Characteristics According to Dietary Patterns

The characteristics of the participants across the quartile of the four dietary patterns are presented in Table 3. Participants in the highest quartile of the rice-vegetable pattern were more likely to be older, male, current smoker, and alcohol drinker; have lower level of education; and exhibit a higher proportion of sleeping disorders, higher energy intake, abdominal obesity, and higher intake of salt than those in the lowest quartile (*P* <0.05). Participants with higher adherence to the fast food pattern tended to be younger, had higher levels of education; and had more sedentary time (*P* <0.001). In addition, there were also significant differences in the family history of hypertension and sleeping disorders across the fast food pattern (*P* <0.05). Participants in the highest quartile of the fruit-dairy pattern were more likely to be younger, female, have higher level of education, never smoker, no drinking, and exhibit significantly lower WC and prevalence of hypertension than those in the lowest quartile (*P* <0.05). Participants in the highest quartile of the wheat-meat pattern were more likely to be younger, male, and without hypertension than their counterparts in the lowest quartiles (*P* <0.001). Moreover, there were significant differences in sleeping disorders, level of education, and family history of hypertension across the quartile of the wheat-meat pattern (*P* <0.05).

**Table 3:** Sociodemographic, lifestyle, and anthropometric characteristics of the study participants across quartiles of the dietary patterns scores (*n* = 2,718).

	**Rice–vegetable**		**Fast food**		**Fruit–dairy**		**Wheat–meat**	
	**Q1**	**Q4**	**Q1**	**Q4**	**Q1**	**Q4**	**Q1**	**Q4**
Age	41 (33, 58)	51 (37, 62)**	54 (38, 64)	39 (32, 56)**	52 (36, 63)	43 (33, 59)**	54 (38, 64)	41 (34, 57)**
Male (%)	269 (39.6)	414 (61.0)**	337 (49.6)	345 (50.8)	402 (59.2)	244 (35.9)**	299 (44.0)	392 (57.7)**
Education (%)								
Low	93 (13.7)	112 (16.6)**	134 (19.8)	75 (11.1)**	142 (20.9)	72 (10.7)**	137 (20.3)	89 (13.2)**
Medium	115 (16.9)	184 (27.3)	183 (27.0)	118 (17.5)	185 (27.3)	122 (18.1)	186 (27.6)	128 (19.0)
High	471 (69.4)	379 (56.2)	360 (53.2)	482 (71.4)	351 (51.8)	481 (71.3)	352 (52.2)	458 (67.9)
Smoking (%)								
Never smoker	538 (79.2)	452 (67.1)**	472 (70.0)	512 (75.5)	417 (61.5)	573 (84.9)**	497 (73.4)	468 (69.3)
Ex–smoker	31 (4.6)	52 (7.7)	50 (7.4)	31 (4.6)	47 (6.9)	33 (4.9)	44 (6.5)	41 (6.1)
Current smoker	110 (16.2)	170 (25.2)	152 (22.6)	135 (19.9)	214 (31.6)	69 (10.2)	136 (20.1)	166 (24.6)
Alcohol drinking (%)								
Yes	182 (27.0)	249 (37.1)**	216 (32.0)	212 (31.5)	254 (37.6)	167 (24.8)**	221 (32.8)	232 (34.5)
No	492 (73.0)	423 (62.9)	460 (68.0)	461 (68.5)	422 (62.4)	507 (75.2)	453 (62.2)	441 (65.5)
Sleeping disorders (%)								
Yes	251 (37.5)	309 (46.3)*	306 (45.5)	266 (39.9) *	292 (43.8)	294 (44.0)	328 (44.9)	280 (42.0)*
No	419 (62.5)	358 (53.7)	367 (54.5)	401 (60.1)	375 (56.2)	374 (56.0)	343 (51.1)	387 (58.0)
WC (%)								
Normal	414 (61.0)	360 (53.0)**	376 (55.4)	410 (60.4)	380 (56.0)	433 (63.8) *	365 (53.8)	420 (61.9)*
Abdominal obesity	265 (39.0)	319 (47.0)	303 (44.6)	269 (39.6)	299 (44.0)	246 (36.2)	314 (46.2)	259 (38.1)
Family history of hypertension (%)								
Yes	311 (45.8)	351 (51.7)	318 (46.8)	321 (47.3)*	299 (44.0)	347 (51.1)	344 (50.7)	286 (42.1)**
No	262 (38.6)	224 (33.0)	268 (39.5)	239 (35.2)	251 (37.0)	238 (35.1)	216 (31.8)	287 (42.3)
Unknown	106 (15.6)	104 (15.3)	93 (13.7)	119 (17.5)	129 (19.0)	94 (13.8)	119 (17.5)	106 (15.6)
Daily salt intake (g/d)								
<4	246 (36.2)	225 (33.1)*	245 (36.1)	225 (33.1)	235 (34.6)	249 (36.7)	245 (36.1)	242 (35.6)
4–6	212 (31.2)	173 (25.5)	203 (29.9)	184 (27.1)	170 (25.0)	192 (28.3)	174 (25.6)	179 (26.4)
> 6	221 (32.6)	281 (41.4)	231 (34.0)	270 (39.8)	274 (40.4)	238 (35.1)	260 (38.3)	258 (38.0)
Sedentary time (h/d)								
<3	215 (31.7)	271 (39.9)*	273 (40.2)	199 (29.3)**	277 (40.8)	239 (35.2)	247 (36.4)	236 (34.8)
3–6	258 (38.0)	244 (35.9)	253 (37.3)	257 (37.9)	232 (34.2)	241 (35.5)	252 (37.1)	271 (39.9)
> 6	206 (30.3)	164 (24.2)	153 (22.5)	223 (32.8)	170 (25.0)	199 (29.3)	180 (26.5)	172 (25.3)
Hypertension (%)	141 (20.8)	210 (30.9)**	210 (30.9)	137 (20.2)**	223 (32.8)	142 (20.9)**	246 (36.2)	140 (20.6)**
Energy intake (kcal/d)	1,280.7 (1,026.6, 1,596.1)	2,134.0 (1,647.0, 2,820.4)**	1,483.6 (1,153.9, 2,002.6)	1,864.6 (1,495.2, 2,378.4)**	1,574.2 (1,223.5, 2,063.7)	1,764.1 (1,418.6, 2,274.3)**	1,450.1 (1,130.8, 1,909.0)	1,989.6 (1,619.2, 2,464.2)**

Q, quartile; WC, waist circumference. ^*^P for trend <0.05, ^**^P for trend <0.001.

*Data are presented as median (p25, p7 + 5) for continuous measures and n (%) for categorical measures. P–values were determined by chi–square tests for categorical variables and Kruskal–Wallis tests for continuous variables*.

### Associations Between Dietary Patterns and Hypertension

As shown in Table 4, the fruit-dairy pattern was inversely associated with hypertension both in the crude model (*P* for trend = 0.029) and in the model adjusted for age, sex, energy, education, smoking, alcohol drinking, sleeping disorders, daily salt intake, sedentary time, WC, and family history of hypertension (*P* for trend = 0.002). Compared with the lowest quartile of the pattern, participants at the highest quartile had lower odds of hypertension (OR = 0.55; 95% CI: 0.40, 0.75; *P* = 0.002). Meanwhile, the wheat-meat pattern was also inversely associated with hypertension prevalence in crude model and model adjusted confounders (OR = 0.70; 95% CI: 0.51, 0.97; *P* = 0.049). In contrast, we did not observe significant associations between the other two patterns with hypertension (*P* trend > 0.05). In the first two sensitivity analyses, the association between the fruit-dairy pattern and hypertension remained consistently significant. However, this risk association was rendered statistically non-significant between the wheat-meat pattern and hypertension (data not shown). As shown in Supplementary Table S1, the association between the fruit-dairy pattern and hypertension remained significant if the participants with prehypertension were regarded as a separate group (OR = 0.67; 95% CI: 0.53, 0.84; *P* = 0.001).

**Table 4:** Adjusted odds ratio (95% CI) for the association between dietary patterns and hypertension (*n* = 2,718).

	**Quartile (Q) of the dietary pattern scores**				***P–trend***
	**Q1**	**Q2**	**Q3**	**Q4**	
Rice–vegetable					
Model 1	1 (Reference)	1.19 (0.88, 1.59)	1.22 (0.91, 1.63)	1.23 (0.91, 1.65)	0.509
Model 2	1 (Reference)	1.19 (0.88, 1.62)	1.25 (0.91, 1.72)	1.23 (0.86, 1.75)	0.539
Model 3	1 (Reference)	1.91 (0.87, 1.63)	1.22 (0.88, 1.69)	1.13 (0.79, 1.62)	0.629
Fast food					
Model 1	1 (Reference)	1.14 (0.87, 1.48)	1.19 (0.90, 1.57)	1.02 (0.76, 1.37)	0.584
Model 2	1 (Reference)	1.12 (0.85, 1.49)	1.15 (0.86, 1.54)	1.05 (0.77, 1.44)	0.777
Model 3	1 (Reference)	1.14 (0.86, 1.52)	1.13 (0.83, 1.52)	1.08 (0.78, 1.43)	0.810
Fruit–dairy					
Model 1	1 (Reference)	0.94 (0.71, 1.23)	0.95 (0.72, 1.25)	0.67 (0.50, 0.89)	0.029
Model 2	1 (Reference)	0.88 (0.66, 1.17)	0.91 (0.68, 1.22)	0.59 (0.43, 0.80)	0.006
Model 3	1 (Reference)	0.83 (0.62, 1.12)	0.85 (0.63, 1.15)	0.55 (0.40, 0.75)	0.002
Wheat–meat					
Model 1	1 (Reference)	0.74 (0.56, 0.97)	0.65 (0.49, 0.86)	0.62 (0.47, 0.82)	0.003
Model 2	1 (Reference)	0.78 (0.59, 1.04)	0.70 (0.52, 0.94)	0.66 (0.48, 0.91)	0.041
Model 3	1 (Reference)	0.76 (0.57, 1.02)	0.70 (0.52, 0.95)	0.70 (0.51, 0.97)	0.049

*WC, waist circumference.*

*Model 1, adjusted for sex and age. Model 2, further adjusted for energy intake, education, smoking, alcohol drinking, sleeping disorders, daily salt intake, sedentary time, and WC. Model 3, additionally adjusted for family history of hypertension*.

## Discussion

In this study, we observed that the fruit-dairy pattern was independently and inversely correlated with hypertension. No significant associations were found for the other three dietary patterns identified after multivariable adjustments and by sensitivity analyses. The crude prevalence of hypertension in our study was 26.7%, which was at an intermediate level compared with the rates in other areas in China (estimated range: 18.0–44.7%) (25, 26).

Factor analysis deriving dietary patterns in our study yielded simple structure and great interpretability (27). It has been widely used to explore the association between multiple dietary components and chronic diseases, which allows for comparisons in different regions (28).

### The Four Dietary Patterns and Hypertension

The fruit-dairy pattern was associated with a lower risk of hypertension, which was consistent with the previous studies (29, 30). A cohort study conducted in Shanghai suggested that more adherence to a fruit and milk pattern characterized by fruit and milk was associated with a decreased prevalence of both pre-hypertension and hypertension (31). The fruit and milk pattern was similar to the fruit-dairy pattern identified in our study in that fruit and yogurt were the primary components of the pattern. A number of mechanisms may explain this finding.

First, nutrients included in fruits and dairy have been reported to be effective in lowering BP (32, 33). Marques et al. (34) found that a high intake of dietary fiber from fruit has a protective effect on hypertension and other cardiovascular diseases *via* changing gut microbiota. Second, when we examined each individual fruit in the fruit-dairy pattern, a strong positive association was observed for apples and citrus fruits. It has been reported that the consumption of apples and citrus fruits may help to regulate BP (35). Nutrients, including vitamin C, potassium, and magnesium, rich in apples and citrus fruits, may play important roles in BP regulation pathways (36). Indeed, we observed that the participants in the highest quartile of the fruit-dairy pattern were more likely to have higher intakes of vitamin C, potassium, and magnesium than those in the lowest quartile (*P* <0.01). Third, milk and yogurt are rich sources of both calcium and vitamin D, which have been shown to work together in vascular smooth muscle cells to regulate BP through the regulation of intracellular calcium concentrations (37). Fourth, the fruit-dairy pattern can indirectly lower BP *via* body weight regulation, whereas overweight/obese is one of the major risk factors for hypertension (38). A study comprising two cohorts showed that increased intake of fruit was beneficial to mitigate body weight (39). Panahi et al. (40) demonstrated that frequent yogurt consumption meant a healthier diet quality, as consumption of yogurt can effectively control body weight, energy homeostasis, and glycemic level, thus contributing to better metabolic health. Fifth, our study indicates that subjects who belonged to the highest quartile of the fruit-dairy pattern were more likely to be alcohol abstainers and have a normal WC, compared with those in the lowest quartile. Emerging evidence has shown that alcohol drinking and abdominal obesity were closely related to the elevation of BP (41–43).

In our study, the fruit-dairy pattern was a new pattern compared with a previous study conducted in Eastern China in 2016 (15), which implies that the eating habits have been in transition in this area (44). Meanwhile, the fruit-dairy pattern was inversely associated with hypertension, which provides a new direction to prevent and control hypertension among community residents in Eastern China.

The other three patterns were not significantly associated with hypertension in our study. The null association of the rice-vegetable observed in our study was inconsistent with a previous report (15). The potential antihypertensive effect of vegetable consumption may be offset by the detrimental effect of polished rice, oil, and salt use (when stir-frying vegetables in Chinese cuisine) (45). Another explanation is that rice and vegetable in the region may have relatively high levels of lead and cadmium (16, 17), which were related to BP. For the fast food pattern, our finding was supported by a cross-sectional study, including 2,560 Chinese participants, which showed that Western fast food patterns were not associated with hypertension (46). The fast food pattern was similar to the high fast food pattern, which was reported as a risk factor for cardiovascular disease (47), but participants with better adherence to the fast food pattern tended to be younger and had fewer sleeping disorders in our study. Younger and better sleeping quality has been considered as protective factors for BP (48, 49). Moreover, participants in the highest quartile of the fast food pattern were more likely to have higher nuts intake than those in the lowest quartile (*P* <0.001). Nuts are potentially protective against hypertension because of their complex compositional characteristics, such as high amounts of beneficial minerals (calcium, magnesium, and potassium) and low level of sodium (50). The wheat-meat pattern was somewhat comparable with the modern pattern reported by the China Health and Nutrition Survey (51). Although red meat intake was recognized as a risk factor for hypertension, this pattern was inversely associated with the consumption of desserts, another risk factor for BP (52). The participants in the highest quartile of the wheat-meat pattern were more likely to avoid dessert intake than those in the lowest quartile (*P* <0.05).

### Strengths and Limitations

The main strengths of this study include its representative sample of the residents in Suzhou, Eastern China, and the latest evidence on dietary patterns and hypertension, which could provide new insight into the prevention of hypertension in this area. However, limitations also need to be considered in the interpretation of our findings. First, bias may exist for dietary data collected by a 24-h dietary recall. A 24-h dietary recall may not be able to well reflect long-term eating habits among the participants (53). Second, we were unable to assess the salt intake using 24-h urine sodium excretion considered as the least biased method, since urine samples were not collected (54). Third, although a wide range of sociodemographic and health-related variables were included as potential covariates in this study, residual confounding may still exist. Fourth, the cross-sectional design of this study limited causal inference, and reverse causation cannot be completely precluded.

## Conclusion

This study provided the latest evidence on the association between dietary patterns and hypertension among community residents in Eastern China. Four dietary patterns were derived in this study by using factor analysis. We observed that the fruit-dairy pattern was inversely associated with the risk of hypertension among the study participants. We suggest that it is necessary to consider the whole diet when making hypertension prevention recommendations for policy-makers. Also, more prospective studies are warranted to determine the relationship between dietary quality and hypertension in different areas in China.

## Data Availability Statement

The raw data supporting the conclusions of this article will be made available by the authors, without undue reservation.

## Ethics Statement

The studies involving human participants were reviewed and approved by Ethics Committee of Suzhou Center for Disease Control and Prevention (SZJK2018-YY001). The patients/participants provided their written informed consent to participate in this study.

## Author Contributions

CW formulated the research questions. YZhe and CW analyzed the data, interpreted the findings, and wrote the manuscript. YZha, DL, and LG compiled the drawings, proofread, and corrected the original manuscript. BW and HZ designed the study, supervised the work, critically revised the manuscript, and approved the final manuscript. All authors contributed to the article and approved the submitted version.

## Conflict of Interest

The authors declare that the research was conducted in the absence of any commercial or financial relationships that could be construed as a potential conflict of interest.

## Publisher's Note

All claims expressed in this article are solely those of the authors and do not necessarily represent those of their affiliated organizations, or those of the publisher, the editors and the reviewers. Any product that may be evaluated in this article, or claim that may be made by its manufacturer, is not guaranteed or endorsed by the publisher.
