# Supplementary material for: Dietary Patterns in Association With Hypertension: A Community-Based Study in Eastern China
Source: Front Nutr. 2022 Jul 8;9:926390. doi: 10.3389/fnut.2022.926390 (PMC9305172; doi:10.3389/fnut.2022.926390)
Supplement: Supplementary file 1 [file Table_1.DOCX]

**Table S1.** Adjusted odds ratio (95% CI) for the association between the dietary patterns and hypertension (n=2718).

|  | Quartile (Q) of the dietary pattern scores | | | | *P trend* |
| --- | --- | --- | --- | --- | --- |
|  | Q1 | Q2 | Q3 | Q4 |  |
| Rice-vegetable | 1 (Reference) | 1.09 (0.87, 1.36) | 1.06 (0.84, 1.33) | 0.91 (0.71, 1.18) | 0.475 |
| Fast food | 1 (Reference) | 0.98 (0.78, 1.21) | 1.13 (0.91, 1.41) | 1.06 (0.84, 1.33) | 0.557 |
| Fruit-dairy | 1 (Reference) | 1.00 (0.80, 1.24) | 0.80 (0.64, 1.00) | 0.67 (0.53, 0.84) | 0.001 |
| Wheat-meat | 1 (Reference) | 0.86 (0.69, 1.07) | 0.85 (0.68, 1.06) | 0.83 (0.62, 1.05) | 0.354 |

WC, waist circumference.

Models were adjusted for sex, age, energy, education, smoking, alcohol drinking, sleeping disorders, daily salt intake, sedentary time, WC and family history of hypertension.
